# The Effect and Associate Mechanism of Nano SiO_2_ Particles on the Diffusion Behavior of Water in Insulating Oil

**DOI:** 10.3390/ma11122373

**Published:** 2018-11-26

**Authors:** Wenxin Tian, Chao Tang, Qian Wang, Shiling Zhang, Yali Yang

**Affiliations:** 1 College of Engineering and Technology, Southwest University, Chongqing 400715, China; 13657601997@163.com (W.T.); Z372475575@163.com (Y.Y.); 2 Electric Power Research Institute of State Grid Chongqing Electric Power Company, Chongqing 401123, China; wangq239@163.com (Q.W.); 13243524193@163.com (S.Z.)

**Keywords:** insulating oil, molecule simulation, nano-SiO_2_, free volume, interaction energy, diffusion coefficient

## Abstract

Moisture has a significant effect on the internal insulation performance of transformers, and is closely related to the breakdown voltage of transformer insulating oil. In the present work, we studied the effect of nano-SiO_2_ particles on the diffusion of water in insulating naphthenic mineral oil using molecular dynamics simulation. Six models were established, three of which contained nano-SiO_2_ particles together with water concentration of 1 wt.%, 2 wt.%, or 3 wt.%. For each model variations in free volume, mean square displacement, and interaction energy were assessed. The addition of nano SiO_2_ particles was found to reduce the free volume fraction of the model and as well as the free motion of water molecules in the oil. These particles also increased the interaction between the oil and water molecules, indicating that insulating oil containing nano-particles has a greater binding effect on water. The diffusion coefficient of water in oil containing nano-SiO_2_ particles was reduced, such that water molecules were less likely to diffuse. The results also show that these particles adsorb water molecules in the oil and to reduce diffusion. Consequently, the addition nano-scale SiO_2_ particles could potentially improve the breakdown voltage of the insulating oil.

## 1. Introduction

With the rapid development of China’s power industry, transformers have become vital components of the power transmission system. The internal insulation of these transformers is based on oil that gradually deteriorates due to many factors during prolonged use, resulting in a decrease in the insulating performance of the oil. This loss of performance is partly associated with increased water levels in the oil, together with other impurities. In the presence of an electric field, the so-called ‘small bridge’ effect of such impurities will render the insulating oil more vulnerable to breakdown [1,2,3].

Nano-particles have a variety of special properties [4] because of their small sizes and large specific surface areas. In addition, nano-particles do not readily generate a ‘small bridge effect’ [5] even in the presence of an electric field. Therefore, the use of nano-particles to improve the performance of insulating oils has become a research topic of significant interest.

There have been many studies in which different nano-particles have been added to insulating oil to explore the effect on the performance of the oil. Du et al. [6] added nano-TiO_2_ to oil and reported that the breakdown voltage of the oil was increased by a factor of 1.15. Katiyar et al. [7] found that a maximum breakdown voltage of approximately 68 kV was achieved when nano-scale Al_2_O_3_ was included in insulating oil at 0.25 wt.%. In addition, both nano-C_60_ [8] and CaCu_3_Ti_4_O_12_ (CCTO) [9] have been widely used in the study of modified insulating oils.

Because SiO_2_ is an insulating semiconductor, nano-SiO_2_ particles can mitigate the challenges associated with magnetic nano-materials that are easily affected by magnetic fields [10]. Li [11] and other researchers have also determined that the viscosity of insulating oil containing nanometer-sized SiO_2_ particles is lowered, based on molecular simulations that assessed the effects of temperature on the viscosity of oil with nano-SiO_2_, Al_2_O_3,_ and ZnO particles. Rafiq et al. [12] added 20% nano-SiO_2_ particles to insulating oil and found that the AC breakdown voltage of the oil increased, although raising the humidity gradually lowered the breakdown voltage. Zhou et al. [13] combined nano-SiO_2_ with insulating oil and showed that the breakdown voltage was considerably improved.

The above studies have demonstrated that the addition of nano-SiO_2_ particles can effectively improve the breakdown voltage of insulating oil. However, although the presence of water is the primary cause of changes in the breakdown voltage of insulating oil, there has been little research regarding the mechanism by which nano-particles affect water diffusion in oil [14,15]. Understanding the water distribution mechanism and the effects of associated factors will therefore be an important aspect of future research regarding nano-modification. Studies have shown that, after 25 years of continuous operation, the moisture content of the average transformer is approximately 3 wt.% [16,17]. Therefore, the present work examined the diffusion behavior of water in nano-doped insulating oil in conjunction with 1 wt.%, 2 wt.% and 3 wt.% water concentrations. The diffusion behaviors associated with varying levels of water in insulating oils are discussed herein, and the mechanisms at work in nano-SiO_2_ modified insulating oils are described on the molecular level. These results should assist in providing a theoretical foundation for further research regarding nano-modified insulating oils.

## 2. Materials and Methods

### 2.1. Model Building

**Figure 1:**
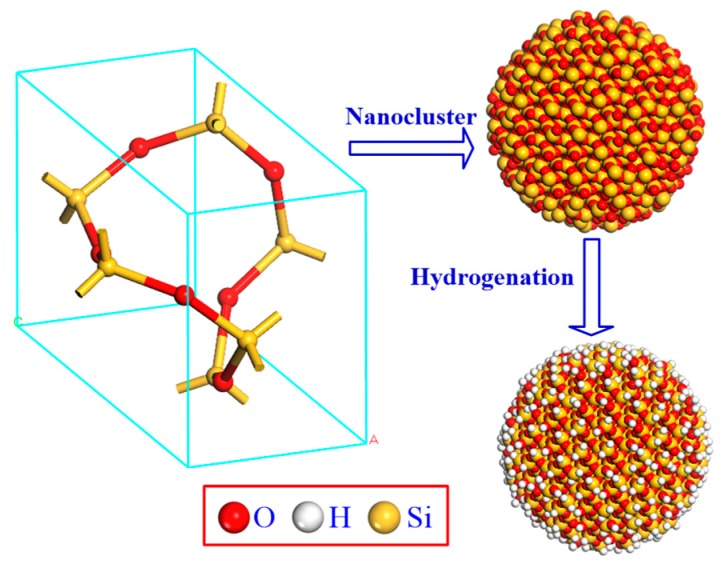
The model for the molecular structure of nano-SiO_2_ particle clusters.

A model of a cluster made of nano-SiO_2_ particles was created using the Materials Studio 5.0 Building Tool software (version) package [18] as shown in Figure 1. After drawing the cluster, hydrogen atoms were added to the unsaturated bonds.

**Figure 2:**
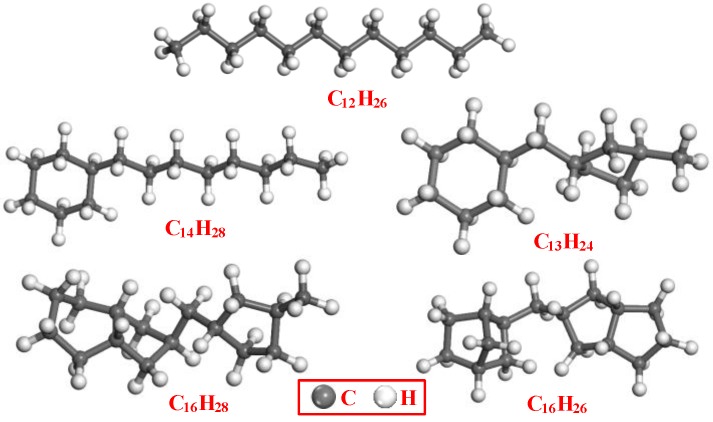
Molecules used during simulations of naphthenic mineral oil.

Mineral oil is widely used in power transformer insulation systems due to its excellent insulating properties and suitable thermal conductivity. Based on the levels of various hydrocarbons, insulating mineral oil is referred to as either paraffin or naphthenic base oil. Naphthene oil is widely used because of its excellent low temperature performance [19]. Thus, in this work, simulations were based on naphthenic mineral oil. The composition of this oil is too complex to allow all the molecular structures to be fully characterized, and so only a limited number of molecules that represent the main physical and chemical properties of the insulating oil were employed in building the model. Figure 2 shows the five alkanes used for this purpose [20], while the mass fraction of each alkane is provided in Table 1.

**Figure 3:**
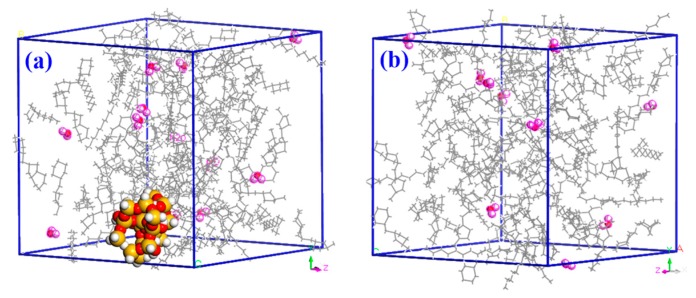
Diagrams of models (**a**) with and (**b**) without nano-SiO_2_.

Based on our goal of modeling varying levels of water in the oil, three groups of two models were constructed using Theodorou’s [21] method of building amorphous polymers. Within each group, the models included an oil/water mixture containing nano-SiO_2_ and another mixture without nano-SiO_2_. The mass-based percentages of water in the three groups of models were 1%, 2%, and 3%. The density of each of the six models was 0.6 g/cm^3^ and the radius of the nano-SiO_2_ particles was 5 Å, the concentration of the nano-SiO_2_ particles was 5 wt.%, with a model size of 4 × 4 × 4 nm [22,23]. Figure 3 presents diagrams of the two models containing 1% water, with and without nano-particles. All MD simulations were carried out using Material Studio.

### 2.2. Simulation Details

**Figure 4:**
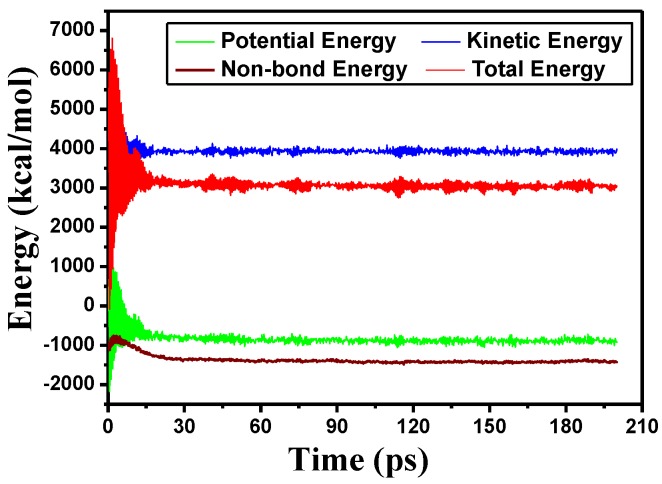
Variations in the energy of simulation models over time.

Prior to molecular dynamics simulations [24], the model was geometrically optimized, after which it was annealed and again geometrically optimized. Those systems that achieved equilibrium and energy convergence after this process could be employed for the molecular dynamics simulation. During these simulations, the model temperature was set to 70 °C [25], because this is the typical operating temperature of a transformer. Initially, the simulation was run over a time span of 200 ps at atmospheric pressure, employing a constant number of particles, pressure, and temperature (that is, an NPT ensemble), plots summarizing variations in the energy of the different models are presented in Figure 4. Subsequent simulations were performed for 300 ps using an NVT (Number of particles, Volume, Temperature) ensemble. The integral step was set to 1 fs and the dynamic information for each atom in the system was collected at 500 fs intervals [26,27].

The COMPASS force field [28] was used during energy optimization and dynamic simulations, while the Nose and Berendsen methods were employed to control the temperature and pressure, respectively. The initial velocity of a particle was randomly assigned according to the Boltzmann distribution; the Velocity Verlet internal algorithm was used. This work also used the Amorphous Cell module to build the model, while the Forcite module was selected for model optimization and dynamic simulation.

## 3. Results

### 3.1. Free Volume

The free volume in the model is an important factor affecting the diffusion behavior of the material, as the free volume provides the necessary active space for small particles. Thus, the size and shape of this volume affects the diffusion behavior of water molecules in the oil [29]. According to the free volume theory of Fox and Flory [30], the total volume of a polymer, V_T_, is composed of the volume occupied by the polymer, V_0_, and the free volume not occupied by the polymer, V_F_. The ratio of free to total volume is the fractional free volume (FFV).

**Figure 5:**
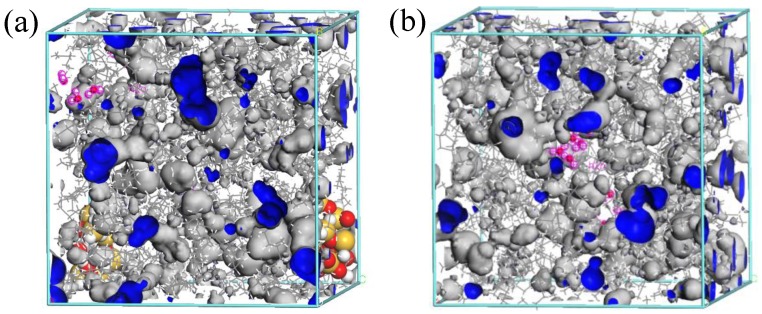
Free volume diagrams of the statistical Connolly surfaces for models (**a**) with and (**b**) without nano-SiO_2_, the blue area is the free volume and the gray area is the occupied volume.

The free volumes of small molecules having different sizes will be different in the same model, and are primarily determined by the properties and sizes of the small molecules. This work employed the Materials Studio Atom Volume and Surface software program to create a Connolly surface, so as to calculate the free volume in the system. A hard ball probe was used, the radius of which equaled the van der Waals radius of a water molecule (1.60 Å). Figure 5 shows the Connolly surface for a two-component model with and without nano-particles, containing 1 wt.% water.

The data in Table 2 demonstrate that the free volume fraction was smaller in those models incorporating nano-particles. In addition, increasing the water level evidently also increased the free volume fraction. These results indicate that the presence of nano-SiO_2_ reduced the diffusion volume available to the water molecules, thus constraining the movement of water in the oil. However, with increasing water concentrations in the oil, the binding of the nano-particles to water molecules was reduced and the free volume fraction increased, allowing more free space for the diffusion of water. As a result, the difference in the free volume fractions of the models with and without nano-SiO_2_ particles was minimized at 3% water. This result demonstrates that the effect of nano-particles on the free volume of the model decreases with increases in the level of moisture.

### 3.2. Interaction Energy

The interaction energy, *E*, between the water molecules and oil is also an important factor affecting the diffusion behavior of the water molecules. This value can be calculated using the Equation (1).

(1) $$E=E_{total}-E_{A}-E_{B},$$

where $E_{total}$ is the total potential energy of the model, and $E_{A}$ and $E_{B}$ are the potential energy values for the oil and water molecules, respectively.

In the model containing nanoparticles, the interaction energy, *E* value for the water molecules and oil can be obtained from the Equation (2) [27].

(2) $$E=\left(E_{total}-E_{A}-E_{B}-E_{A+C}-E_{B+C}+E_{C}+E_{A+B}\right)÷2,$$

where $E_{A+C}$, $E_{B+C}$, $E_{C}$ and $E_{A+B}$ are the total potential energy values of the oil and nano-particles, water molecules and nano-particles, nano-particles, and water molecules and oil, respectively. Table 3 provides the interaction energy values between water molecules and oil in the six different models.

**Figure 6:**
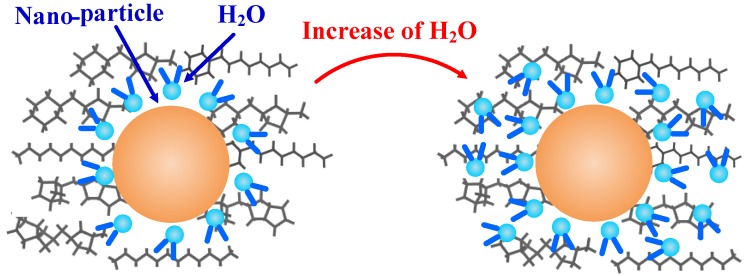
The adsorption of water molecules by nano-particles.

If the interaction energy is positive, the substances do not combine, whereas negative interaction energy indicates that the materials will attract one another [31]. The data in Table 3 demonstrate that, in the case of oil containing nano-particles, the absolute value of the interaction energy is larger than that of the insulating oil model without nano-particles. Therefore, the presence of nano-particles increases the binding force experienced by the water molecules. The adsorption of water molecules by nano-particles is shown schematically in Figure 6. In Figure 6 shows that nano-SiO_2_ particles have a good effect on water molecules at low moisture content, so water molecules are evenly distributed around nano-SiO_2_ particles. However, with the increase of water molecules, the binding of nano-SiO_2_ particles to water gradually weakens, so, it shows that the additional H_2_O is less strictly oriented in the radial direction.

**Figure 7:**
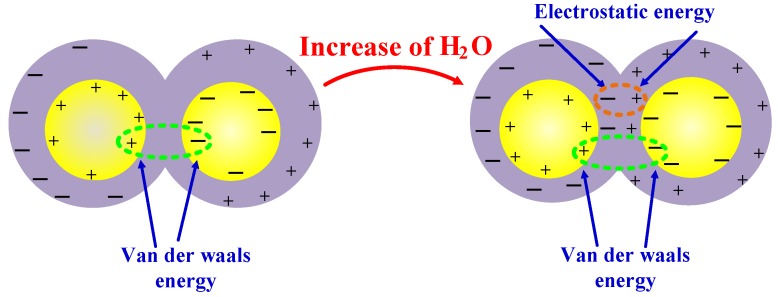
A schematic summarizing the variation of the intermolecular interaction energy.

Because water molecules and oil have very different polarities, they would not be expected to undergo electrostatic interactions, and the interaction energy is primarily due to van der Waals forces. However, in the model containing nano-particles, the electrostatic interaction energy is positive, indicating a significant change in the polarity. In the case of oil without nano-particles, the electrostatic energy is negative, suggesting that the polarity of the oil species has changed. This phenomenon further demonstrates that the nano-particles tend to adsorb water molecules, such that the water molecules do not fully interact with the oil. With increases in the water concentration, the interaction energy between the oil and water molecules increases, meaning that the oil more strongly bonds with the water. This effect is the opposite of the trend predicted by traditional free volume theory. The variations in the interaction energy with composition show that increases in the water concentration increase the polarity of the oil. As a result, there is a gradual increase in the Coulomb effect, the van der Waals forces are reduced, and the oil molecules are gradually polarized. Thus the oil molecules have a greater effect on the polar water molecules. The present research shows that the electrostatic interaction between the oil and the water molecules in the presence of nano-particles is less than that without nano-particles. This occurs because the nano-SiO_2_ particles adsorb water such that the water in the oil has a lesser effect on the polarity of the oil. As a result, the electrostatic attraction between the oil and the water molecules is lessened. Figure 7 presents a schematic diagram that summarizes the effect of increasing the water content on the interaction energy between water and oil molecules.

### 3.3. Mean Square Displacement

**Figure 8:**
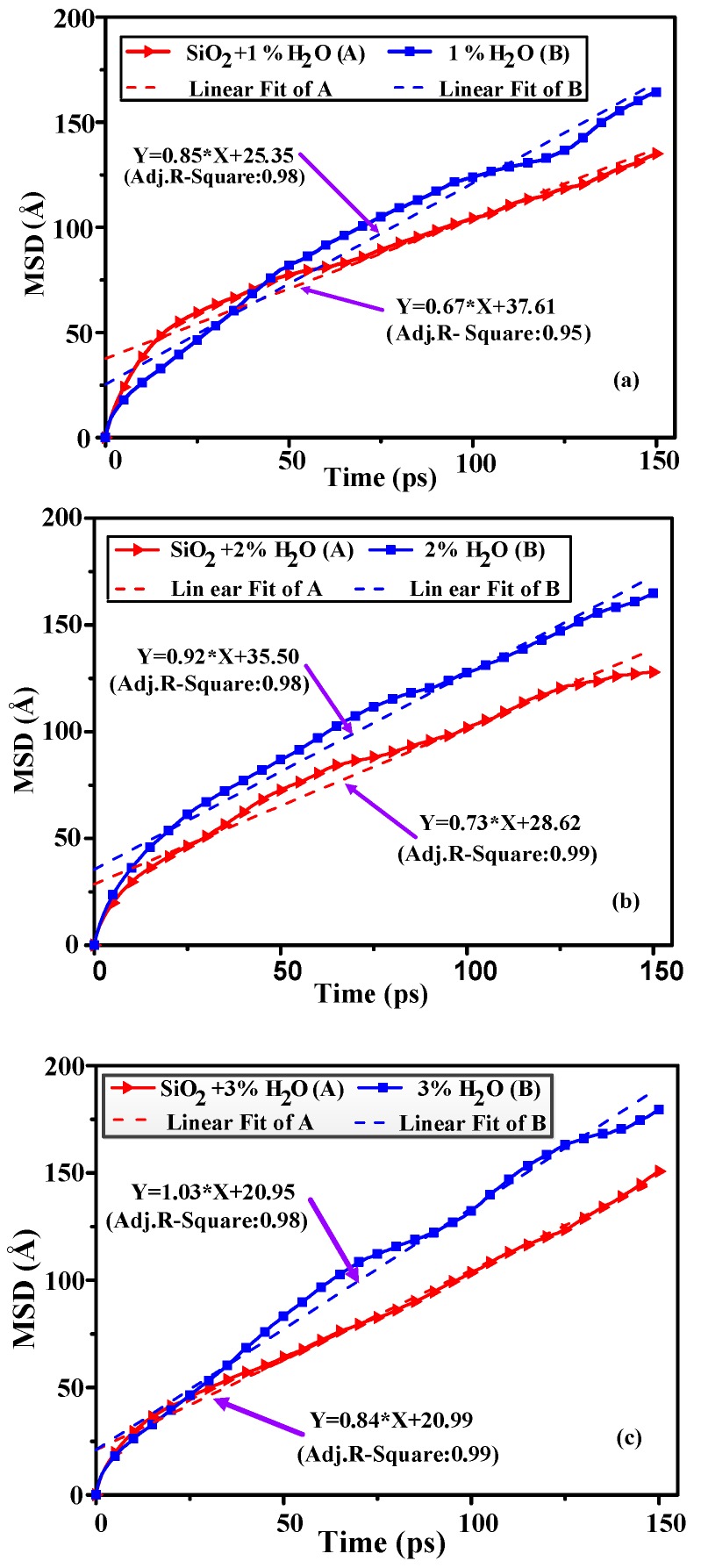
The mean square displacement values of water molecules as functions of time for all six models (**a**) the mean square displacement (MSD) for water molecules of 1 wt.% moisture; (**b**) the MSD for water molecules of 2 wt.% moisture; (**c**) the MSD for water molecules of 3 wt.% moisture.

Mean square displacement (MSD) can study the diffusion behavior of water molecules in insulating oil. The diffusion coefficient is an important parameter that can be used to assess the diffusion capacity of a material. The larger the diffusion coefficient, the smaller the media’s effect on the diffusion of particles. The diffusion coefficient (*D*) can be obtained from the slope of their MSD in a time interval with Einstein relation. This coefficient can be calculated via the Equation (3):

(3) $$D=\frac{1}{6N}\underset{t\to \infty }{lim}\frac{d}{dt}\sum_{i=1}^{N}{\left(r_{i}\left(t\right)-r_{i}\left(0\right)\right)}^{2},$$

where $r_{i}\left(t\right)$ and $r_{i}\left(0\right)$ are position vectors for the ith atom at time *t* and time zero, respectively, and N is the number of water molecules in the model. The MSD for all water molecules in each model are shown in Figure 8. To verify the Einstein relation, linear fit for all data from 0–300 ps, collect data every 2 ps. And since the beginning part of MSD is relatively chaotic, however, from the point of view of goodness of fit, it does not affect the calculation of diffusion coefficient [32].

Table 4 gives the diffusion coefficients of water molecules in the various models as obtained by mean square displacement fitting.

As can be seen from Figure 8, the correlation coefficient for each plot is greater than 0.9, showing reasonable curve fitting. The data demonstrate that, in the case of the model containing nano-SiO_2_ particles, the diffusion coefficient is smaller than in the model without nano-particles. It shows that the diffusion ability of water molecules in insulating oil containing nanoparticles is weaker, which proves that the addition of nanoparticles can effectively restrain the diffusion of water molecules in oil. In addition, increasing the water concentration in the oil raises the diffusion coefficient of water molecules, it shows that with the increase of water content, the diffusion ability of water molecules in oil increases gradually. The classical free volume theory explains the diffusion of small molecules in amorphous polymers. The models without nano-particles show larger free volume fractions that increase with increases in the water level. This effect occurs because greater amounts of free volume provide more space for water molecules, such that the diffusion coefficient of these molecules also increases and the diffusion capacity is improved. This is in accordance with the classical free volume theory. It also provides a basis for nanoparticles to bind water molecules in oil.

## 4. Conclusions

The diffusion behavior of water molecules in nano-modified insulating oil was studied by molecular simulations, concentrating on variations in the free volume, diffusivity and interaction energy at different water levels. On the basis of the results, we present the following conclusions.

(1) The free volume fractions in the models containing nano-SiO_2_ particles were reduced, and so the diffusion of water molecules was restricted. Thus, water molecules had a smaller diffusion coefficient in oils containing nano-particles, meaning less diffusion occurred.
(2) The model containing nano-SiO_2_ particles showed greater interaction energy between the oil and water molecules, demonstrating that the addition of these particles increased the binding of water molecules by the oil.
(3) The results prove that the addition of nano-SiO_2_ particles can effectively increase the binding of insulating oil to water molecules and reduce the diffusion of water molecules in insulating oil. This paper provides a theoretical basis for the modification of insulating oil with nano-SiO_2_ particles.

## Figures and Tables

**Table 1:** Mass-based percentages of various molecules used during simulations of naphthenic mineral oils.

Composition	Chain Hydrocarbon	Cycloparaffins				Total
		Monocyclic	Dicyclic	Tricyclic	Tetracyclic	
ω_B_ (%)	11.6	15.5	28.5	23.3	9.7	88.6

**Table 2:** Summarizes the free volume fractions of models having different water contents (Å^3^). FFV: fractional free volume.

	Nano-SiO_2_ Particles			Without Nano-SiO_2_ Particles		
Moisture	1%	2%	3%	1%	2%	3%
Occupied volume	35,308	35,840	34,914	34,327	34,719	33,692
Free volume	3485	3550	5902	3640	4107	5808
FFV	0.089	0.090	0.145	0.096	0.103	0.147

**Table 3:** The interaction energy of water molecules and oil media (kcal/mol).

	Nano-SiO_2_ Particles			Without Nano-SiO_2_ Particles		
Moisture	1%	2%	3%	1%	2%	3%
Interaction energy	−16.86	−30.50	−32.98	−15.80	−29.89	−30.62
van der Waals energy	−15.59	−28.24	−20.75	−14.91	−27.32	−18.75
Electrostatic energy	0.59	−0.38	−8.09	−0.578	−1.037	−10.22

**Table 4:** Diffusion coefficients of water molecules (Å^2^/s).

	Nano-SiO_2_ Particles			Without Nano-SiO_2_ Particles		
Moisture	1%	2%	3%	1%	2%	3%
*D*	0.11	0.12	0.14	0.14	0.15	0.17
